# Immunogenicity, Efficacy and Twelve-Month Storage Stability Studies of a Lyophilized Rabies mRNA Vaccine

**DOI:** 10.3390/vaccines13070743

**Published:** 2025-07-10

**Authors:** Chen Chen, Dandan Ling, Kai Ji, Liang Tang, Xiaojing Zhang, Xishan Lu, Xuemei Leng, Changyao Tan, Hongchao Wu, Wenqiang Pang, Quanren He, Jerry Zhang, Peng Gao, Xiaotao Wang, Linhui Wang, Bo Ying

**Affiliations:** 1 Suzhou Abogen Biosciences Co., Ltd., Suzhou 215123, China; chen.chen2@abogenbio.com (C.C.); dandan.ling@abogenbio.com (D.L.); liang.tang@abogenbio.com (L.T.); xiaojing.zhang@abogenbio.com (X.Z.); xishan.lu@abogenbio.com (X.L.); quanren.he@abogenbio.com (Q.H.); zhiyi.zhang@abogenbio.com (J.Z.); peng.gao@abogenbio.com (P.G.); xiaotao.wang@abogenbio.com (X.W.); 2 Jiangsu CuroVax Co., Ltd., Changzhou 213032, China; lengxuemei@curovax.net (X.L.); tanchangyao@curovax.net (C.T.); 3 National Research Center for Veterinary Medicine, Luoyang 560119, China; wuhongchao@pulike.com.cn (H.W.); pangwenqiang@pulike.com.cn (W.P.)

**Keywords:** rabies, mRNA vaccine, lipid nanoparticles, lyophilization, long-term stability, beagles

## Abstract

**Background:** Many new mRNA-based vaccine candidates in liquid mRNA-LNP formulations are under development; however, their stability limitations necessitate frozen storage, posing a significant challenge for long-term storage and transportation. **Methods:** In this study, an mRNA-LNP rabies vaccine, ABO1005, was prepared, freeze-dried and stored at 2–8 °C for 12-month storage stability evaluation. The immunogenicity, vaccine potency (the NIH method), and protective efficacy of ABO1005 were assessed in mice or dogs and compared to a commercialized inactivated vaccine. **Results:** Research conducted in mice indicated that the lyophilized vaccine exhibited comparable immunogenicity to its liquid form counterpart. Furthermore, the vaccine candidate elicited a robust humoral response lasting at least 175 days, and the specific antibody titers were not affected by the pre-administration of hyperimmune serum. In comparison to the commercialized inactivated vaccine (HDCV or PVRV), ABO1005 elicited significantly higher levels of humoral and cellular immunity. Vaccine potency testing (NIH) revealed that the potency of ABO1005 at 15 μg/dose was 8.85 IU/dose, which is substantially higher than the standard required for the lot release of rabies vaccines for current human use. In a post-exposure prophylaxis (PEP) study in Beagle dogs, the lyophilized vaccine provided 100% protection for dogs following a two-dose regimen (D0-D7), whereas commercially approved inactivated vaccine offered 83% protection. After storage at 2–8 °C for 12 months, no notable changes were observed in the particle size, encapsulation efficiency, and integrity of mRNA or in the immunogenicity of the lyophilized vaccine. **Conclusions:** This study successfully developed a formulation and process of freeze-drying for a rabies mRNA vaccine, paving the way for future lyophilized mRNA vaccine development.

## 1. Introduction

Rabies is a life-threatening zoonosis with a mortality rate approaching 100% among infected individuals who exhibit neurological symptoms, such as coordination disorders, convulsions, and paralysis [1]. The rabies virus (RABV) is classified within the genus *Rhabdovirus* of the *Rhabdoviridae* family. Its surface spike protein, known as glycoprotein (RABV-G), is responsible for the induction of rabies viral neutralizing antibodies (VNAs) in vivo [2,3]. The RABV-G precursor consists of a signal peptide (SP), an extracellular domain (ECD), a transmembrane domain (TM) and an intracellular domain (ICD), and it is believed to assemble into homo-trimers on the viral surface, and the trimeric pre-fusion form has the epitopes for neutralizing antibodies [2,4].

Currently, the inactivated rabies vaccine (IRV), manufactured using various cell types, including the human diploid cells, African green monkey kidney (Vero) cells, primary chick embryo cells, and primary hamster kidney cells [5,6], has played a critical role in protecting humans through post-exposure prophylaxis (PEP) [7]. The IRV primarily elicits antibody responses with limited or no cellular responses, which may be essential for protection during PEP [8]. Therefore, unvaccinated individuals seeking protection must complete the full PEP course as soon as possible. This course entails either the Zagreb regimen (2-1-1) within 21 days or the Essen regimen (1-1-1-1-1) within 28 days, as necessary, while rabies immunoglobulin is concurrently administered [9]. However, frequent visits for vaccination may confuse recipients and result in decreased compliance. Notably, over one-quarter of all clinical trials involving rabies conducted over the past decade have aimed to test accelerated vaccination schedules [10]. During the development of COVID-19 and varicella-zoster virus (VZV) vaccines, mRNA formulations have shown strong innate and cellular immunity as the mRNA has a self-adjuvant effect. Consequently, rabies mRNA vaccines are believed to have some advantages over IRV. In a proof-of-concept study in human volunteers, two doses of CV7202 elicited effective VNA responses that met the World Health Organization (WHO) criteria for the titer that adequate to prevent disease and death (VNA ≥ 0.5 IU/mL) in all recipients [11]. In preclinical studies, two doses of LVRNA001 produced a robust immune response in both mice and dogs and effectively protected exposed dogs against infection [8]. Additionally, two doses of the RABV-G mRNA vaccine induced VNA lasted longer than the IRV [12]. Therefore, rabies mRNA vaccines may present an opportunity for a shorter vaccination regimen compared to the IRV.

To date, approved mRNA vaccines require ultra-cold storage and present significant challenges in terms of transportation and storage costs. The long-term storage of mRNA-LNP vaccines without freezing remains a challenge. However, lyophilized mRNA-LNP vaccines, produced through the removal of water and oxygen, have the potential to improve thermostability and facilitate long-term storage at 2–8 °C.

In this study, we evaluated the effectiveness of a codon-optimized RABV-G mRNA sequence encapsulated in lipid nanoparticles (LNP), designated as ABO1005, in both mice and dogs. The results demonstrate that ABO1005 induced higher level of VNA titers compared to the IRV in mice and protected the vaccinated mice from intracerebral (I.C.) RABV infection. In a PEP study, the lyophilized ABO1005 provided complete protection for dogs against the RABV street strain BD06 and exhibited superior protective efficacy to the commercialized IRV. Notably, the lyophilized ABO1005 demonstrated a long-term stability of at least twelve months at 2–8 °C.

## 2. Materials and Methods

### 2.1. Cell Lines, Antibodies, Vaccines, Viruses, and Animals

HEK293F cells (CAT. NO. R79007, Thermo Fisher Scientific, Waltham, MA, USA) were cultured in suspension culture Expi293^TM^ Expression Medium (CAT. NO. A1435101, Gibco, Grand Island, NE, USA) maintained at 37 °C in a CO_2_ shaking incubator. HEK293T cells (CAT. NO. SCSP-502, National Collection of Authenticated Cell Culture, Shanghai, China) were cultured in complete Dulbecco’s modified Eagle’s medium supplemented with 10% (*v*/*v*) fetal bovine serum (FBS, Gibco). The rabies-neutralizing human antibody, RVA122, was isolated from a vaccinated individual and specifically bound to the trimeric prefusion conformation of the RABV-G [13]. We constructed a fully recombinant human antibody, RVA122-IgG1. The heavy chain variable region of RVA122 was fused to the human IgG1 heavy-chain constant region, while the light-chain variable region of RVA122 was linked to the human lambda light chain (Sino Biological, Beijing, China).

The freeze-dried rabies vaccines for human use including the Human Diploid Cell Vaccine (HDCV^®^, Chengdu Kanghua Biological Products Co., Ltd., Chengdu, China) and the Purified Vero-Cell Rabies Vaccine (PVRV) Chengda Suda^®^ (Liaoning Chengda Co., Ltd., Shenyang, China) are derived from the Pitman-Moore (PM) strain and the Pasteur virus (PV) strain, respectively. Both vaccines were labeled with NIH titers of no less than 2.5 IU per human dose.

The RABV street strain BD06 (GenBank: ACB38373.1) is responsible for the major portions of the rabies cases in both humans and dogs in China and was preserved by the Institute of Military Veterinary Medicine, Academy of Military Medical Sciences (Changchun, China) [14].

Specific pathogen-free (SPF)-grade BALB/c or KM mice (6–8 weeks old) were supplied by Zhejiang Vital River Laboratory Animal Technology Co., Ltd., and were housed at the Suzhou Institute of Systems Medicine (ISM), the Chinese Academy of Medical Sciences.

Rural dogs were sourced from farmers in the vicinity of Luoyang City (Luoyang, China). The feeding, immunization, and blood collection of the dogs were conducted at the animal facility of National Research Center for Veterinary Medicine (Luoyang, China). Beagle dogs were obtained from Jinan Jinfeng Experimental Animal Co., Ltd., and housed in a Biosafety Level 3 (BSL-3) laboratory at the Institute of Military Veterinary Medicine of Academy of Military Medical Sciences (Changchun, China). All dogs used in the experiment were pre-screened to be negative for canine distemper virus, canine parvovirus, canine adenovirus, and canine parainfluenza virus. Additionally, their rabies virus neutralizing antibody titers were below 0.5 IU/mL, and they showed normal mental status, appetite, and fecal condition, along with smooth coats. The protocols for the use of animals and the associated procedures were approved by the animal care and use committee of the respective institutions (see the Institutional Review Board Statement Section).

### 2.2. mRNA Preparation and LNP Formulation

The ABO1005 mRNA sequence was derived from the Pitman-Moore (PM) strain (GenBank: DQ099525.1). The in vitro transcription (IVT) reaction utilizes a linearized DNA template derived from plasmids, with genes synthesized by GenScript Incorporation. This template incorporates the codon-optimized RABV-G sequence, along with the 5′ and 3′ untranslated regions (UTRs) and a poly (A) tail. For mRNA synthesis, the IVT reaction was conducted using T7 RNA polymerase as previously described, with a minor modification [15].

The final mRNA-LNP formulations were prepared using a modified procedure based on a previously described method for mRNA vaccines [16]. Briefly, lipids were dissolved in ethanol containing an ionizable lipid, 1, 2-distearoyl-sn-glycero-3-phosphocholine (DSPC), cholesterol and PEG-lipid (with molar ratios of 50:10:38.5:1.5). The lipid mixture was subsequently combined with a 10–50 mM citrate buffer (pH 3.0–6.0) containing mRNA through a T-mixer. The formulations were then diafiltrated against 1× PBS (pH7.4) or lyophilization buffers (20 mM NaCl, 8 mM K_2_HPO_4_/K_2_HPO_4_, 10% sucrose, pH 5.8), and afterwards, LNP was passed through a 0.22 μm filter. The mRNA concentration, integrity, particle size and encapsulation efficiency were tested for quality control [17]. The mRNA concentration was measured with High-Performance Liquid Chromatography (HPLC). mRNA integrity was determined with the Fragment Analyzer (Agilent). Particle size was determined by dynamic light scattering (DLS) using a Malvern Zetasizer Nano ZS (Malvern UK) with a 173^°^ backscatter detection mode. The encapsulation efficiency (EE %) of lipid nanoparticles was determined using a Quant-it RiboGreen RNA quantification assay kit (Thermo Fisher Scientific, UK), according to the manufacturer’s instructions.

Lyophilization was conducted using a pilot-scale freeze dryer (Epsilon 2-10D LSCplus, Martin Christ, Osterode am Harz, Germany). The mRNA-LNP vials were frozen at temperatures ranging from −40 to −60 °C for 3 h, followed by a primary drying cycle at −25 to −35 °C under a vacuum of 20 to 100 mTorr for 48 to 60 h. During the subsequent secondary drying cycle, the vials were warmed to a temperature range of 5 to 25 °C under 20 to 100 mTorr and further held for 12 to 24 h. At the end of the cycle, all rubber stoppers on the vials were fully sealed under vacuum and capped with flip-off seals before being transferred for long-term storage at 2 to 8 °C.

### 2.3. Cryo-EM

Liquid or reconstitute LNPs were buffer exchanged to 1× PBS and concentrated to 0.8–1 mg/mL. LNP (4 μL) was deposited on a holey carbon grid that was glow-discharged (Quantifoil R1.2/1.3) and vitrificated using a Vitrobot Mark IV (Thermo Fisher Scientific) instrument. Cryo-EM imaging was conducted on a cryo-transmission electron microscope (Glacios-2 Cryo-TEM, Thermo Fisher), operated at 200 kV accelerating voltage.

### 2.4. ELISA

The RABV-G-specific IgG antibodies were detected using an indirect enzyme linked immunosorbent assay (ELISA) [18]. The recombinant RABV-G of PM strain was expressed in insect cells at Sino Biological, Inc. (Beijing, China). Flat-bottom microplates were coated with 100 ng RABV-G per well at 4 °C overnight. The microplates were washed three times with the phosphate-buffered sodium with Tween-20 (PBST, pH 7.4) and subsequently blocked with 5% (*w*/*v*) BSA in PBST at 37 °C for 1 h. After three washes, 100 μL/well of serially diluted serum samples or standard serum (the limiting dilution method–calibrated mouse hyperimmune serum) were added and then incubated at 37 °C for 1 h. After three washes, 100 μL/well of 1:10,000 diluted peroxidase-conjugated anti-mouse IgG (CAT. NO. 715-035-151, Jackson ImmunoResearch, West Grove, PA, USA) was added and then incubated at 37 °C for 1 h. Following three washes, 100 μL/well of 3,3′,5,5′-Tetramethylbenzidine (TMB) substrate (CAT. NO. PR1200, Solarbio, Beijing, China) was added and then incubated at room temperature for a color reaction, which was stopped by the addition of 50 μL/well of stop solution. Finally, the optical density (OD) values at 450 nm for all wells were measured using a SpectraMax iD5 (Molecular Devices, Sunnyvale, CA, USA).

### 2.5. Western Blot Analysis

The RABV-G was identified using Western blotting (WB) [19]. Briefly, the HEK293T cells in 6-well plate were transfected with 2.5 μg of ABO1005 mRNA, ABO1005 or the positive control plasmid (pcDNA3.1-RABVG-PM) using Lipofectamine 2000 reagent (Life Technologies, Carlsbad, CA, USA). At 44–48 h post transfection, the cell monolayer was washed with PBST buffer and lysed using 1% NP-40 buffer (CAT. NO. N8032, Solarbio) containing a complete™ Protease Inhibitor Cocktail (CAT. NO. 11697498001, Roche, Basel, Switzerland). After centrifugation, the supernatants of cell lysates were mixed with 5 × protein loading buffer (CAT. NO. P0015, Beyotime, Shanghai, China) and loaded onto an ExpressPlus™ PAGE Gel (CAT. NO. M41210C, GenScript, Nanjing, China). Following separation via sodium dodecyl sulfate–polyacrylamide gel electrophoresis (SDS-PAGE), the proteins were transferred onto a polyvinylidene fluoride (PVDF) membrane using the iBlot™ 2 Dry Blotting System (Invitrogen, Carlsbad, CA, USA) at 15 V. The membrane containing the transferred proteins was incubated with QuickBlock buffer (CAT. NO. P0252, Beyotime) at room temperature for 1 h. After five washes with PBST, the transferred proteins were probed with a 1:1000 dilution of mouse anti-RABV-G mAb 3E7 (CAT. NO. DMABTZ59957, Creative Diagnostic, Shirley, NY, USA) at 4 °C overnight. Following five washes, a 1:10,000 dilution of peroxidase-conjugated anti-mouse IgG (CAT. NO. 715-035-151, Jackson ImmunoResearch, West Grove, PA, USA) was added and incubated with the membrane at room temperature for 1 h. Afterward, the membrane was incubated with BeyoECL Star (CAT. NO. P0018AS, Beyotime), and protein visualization was performed using the Chemiluminescent Imaging System (Tanon 5200, Tanon, Shanghai, China).

### 2.6. Flow Cytometry Assay for Detection of the Expression of RABV-G

The HEK293F cells (1 × 10^6^ cells/well) was seeded into 24-well culture plate. Following the manufacturer’s instruction, 0.5 μg of ABO1005 mRNA or plasmid (pcDNA3.1-RABVG-PM, positive control) were transfected into the HEK293F cells using Lipofectamine 2000 reagent (Life Technologies). At 20–24 h post transfection, the HEK293F cells were resuspended in FACS buffer (pH 7.4 PBS containing 2% FBS, *v*/*v*). After centrifugation, the cells were resuspended in the 1:1000 diluted mouse anti-RABV-G monoclonal antibody 3E7 (CAT. NO. DMABTZ59957, Creative Diagnostic) and were incubated at 4 °C for 30 min. After three washes, the cells were resuspended in 1:1000 diluted FITC-conjugated anti-mouse IgG (CAT. NO. 115-095-164, Jackson ImmunoResearch) and were incubated at 4 °C for 30 min. After three washes, the cells were resuspended in 200 μL FACS buffer for detection of RABV-G in vitro using a flow cytometry instrument (LSRFortessa™, BD Biosciences, Franklin Lakes, NJ, USA).

### 2.7. Flow Cytometry Assay for Intracellular Cytokine Staining (ICS)

To detect T-cell activation, the mice were vaccinated with ABO1005 on days 0 and 14 and were euthanized following CO_2_ anesthesia on day 7 after the second immunization. The spleens were isolated and ground with syringe push handle, and the resulting cell suspension was filtered through 70 μm pore strainers (CAT. NO. 130-095-823, Miltenyi Biotec, Bergisch Gladbach, Germany). The red blood cells were lysed through a 3 min incubation with lysis buffer (CAT. NO. 555899, BD Biosciences). After two washes with complete RMPI 1640 medium, the cells were filtered through 70 μm pore strainers again. Then, 100 μL of cell suspension per well was added to the 96-well culture plate and stimulated with 100 μL of peptide pool (99 peptides, with each peptide containing 2 μg/mL). The peptide pool covered the full-length RABV-G of PM strain (GenBank: DQ099525.1), with each peptide being 15 mers with 10 amino acid overlap. After stimulation for 4 h, brefeldin A (BFA) was added to the culture medium. After incubation for 16–20 h, the splenic cells were blocked with CD28 (CAT. NO. 122002, BD Bioscience) and CD49d (CAT. NO. 103630, BD bioscience) antibodies. Following staining with Zombie Green Dye (CAT. NO. 423112, Biolegend, San Diego, CA, USA), the splenic cells were washed and resuspended in an antibody cocktail that included AF700 anti-mouse CD3 (CAT. NO. 100216, Biolegend), PerCP-CY5.5 anti-mouse CD4 (CAT. NO. 100434, Biolegend), APC-CY7 anti-mouse CD8a (CAT. NO. 100714, Biolegend), BV711 anti-mouse IFN-γ (CAT. NO. 505836, Biolegend), APC anti-mouse IL-2 (CAT. NO. 503810, Biolegend), BV421 anti-mouse TNF-α (CAT. NO. 506328, Biolegend), and PE-CY7 anti-mouse IL-4 (CAT. NO. 504118, Biolegend) and incubated at 4 °C for 30 min. After three washes, the cells were resuspended in 200 μL of FACS buffer for the detection of activated T cells with the flow cytometry instrument (LSRFortessa™, BD Biosciences).

For the analysis of follicular helper T (Tfh) and germinal center B (GCB) cells in spleens, splenic cells were first blocked with mouse Fc block reagent (CAT. NO. 553141, BD Bioscience), followed by staining with Zombie Green Dye (CAT. NO. 423112, Biolegend). After three washes, the splenic cells were resuspended in an antibody cocktail that included BV605 anti-mouse CD3 (CAT. NO. 100237, Biolegend) and PerCP-CY5.5 anti-mouse CD4 (CAT. NO. 100434, Biolegend) for identifying T cells; AF700 anti-mouse CD19 (CAT. NO. 115528, Biolegend) and BV510 anti-mouse IgD (CAT. NO. 405723, Biolegend) for identifying B cells; PE-CY7 anti-mouse CD95 (CAT. NO. 152618, Biolegend) and APC anti-mouse PD-1 (CAT. NO. 135210, Biolegend) for identifying Tfh cells; and Biotin anti-mouse CD185 (CAT. NO. 145510, Biolegend) followed by BV421 streptavidin (CAT. NO. 405225, Biolegend) and the PE anti-MU/HU GL7 Antigen (CAT. NO. 144608, Biolegend) for identifying GCB cells. The mixture was incubated for 30 min at 4 °C. After three washes, the cells were resuspended in 200 μL/well of FACS buffer per well for the detection of Tfh and GCB cells using the flow cytometry instrument (LSRFortessa™, BD Biosciences). On day 21 post initial immunization, splenic cells were analyzed by gating on either CD3^+^ CD4^+^ CD19^−^ T cells or CD19^+^ CD3^−^ IgD^−^ B cells. Follicular T helper (Tfh) cells, characterized as CXCR5^+^ PD-1^+^ cells, were gated from the T cell population, while germinal center B (GCB) cells, identified as GL7^+^ CD95^+^ cells, were gated from the B cells population.

### 2.8. ELISpot

The enzyme-linked immunospot (ELISpot) assay was conducted as previously described with minor modifications [20]. Briefly, the commercialized mouse IFN-γ ELISpot kit (CAT. NO. 3321-4HST-2, MabTech, Nacka, Sweden) and the mouse IL-2 kit (CAT. NO. 3441-4APW-2, MabTech) each contain pre-coated plates with IFN-γ and IL-2 antibodies, respectively. The pre-coated plates were initially blocked with complete RPMI-1640 medium (10% FBS) for 30 min in a 37 °C incubator. Splenocytes of mice were then plated at a density of 2 × 10^5^ cells per well and stimulated with an RABV-G peptide pool (2 μg/mL of each peptide). A cell stimulation cocktail (CAT. NO. 00-4970-93, eBioscience, San Diego, CA, USA) was included as a positive control and RPMI 1640 media was used as negative control. After incubation at 37 °C in a CO_2_ incubator for 36 h, plates were washed with PBST buffer, and the biotinylated anti-mouse IFN-γ, IL-2 and IL-4 antibodies were added to each corresponding ELISpot plate followed by incubation for 2 h at room temperature. After five washes, 100 μL of substrate solution per well was added for a color reaction. Following five washes with ultrapure water, the plates were air-dried, and the air-dried plates were read using the S6 Universal M2 ELISpot Reader (Cellular Technology Ltd., Cleveland, OH, USA). The number of spot-forming cells (SFC) per 1 × 10^6^ cells was calculated and presented.

### 2.9. Virus Neutralization Titer Assay

RABV-specific VNA titers were measured by the fluorescent antibody virus neutralization (FAVN) test or the rapid fluorescence focus inhibition test (RFFIT), as previously described [20]. The sera were separated and inactivated at 56 °C for 30 min. The FAVN test was conducted in the 96-well plate, and 100 μL of DMEM per well was added to each well, followed by the addition of 50 μL of inactivated test serum or positive reference serum in quadruplicate to the first column. After three-fold dilution, the serum was neutralized with 100 × TCID_50_ of the rabies challenge virus (CVS-11) per well for 1 h at 37 °C. Following incubation, BSR cells at a density of 2 × 10^4^ cells per well were added to each well. After 48 h of incubation at 37 °C in a 5% CO_2_ incubator, cells were then fixed using 80% ice-cold acetone at room temperature for 30 min and stained with FITC-conjugated RABV N-protein antibodies at 37 °C for 1 h. After rinsing, the plate was read using a fluorescence microscope. A well was considered positive if one or more fluorescent cells were observed, while a well was deemed negative if no fluorescent cells were present [21]. Reference serum was obtained from the National Institute for Biological Standards and Control, Hertfordshire, UK.

The RFFIT was conducted with multi-chambered tissue culture slides, and the sera were tested as quadruplicates in 3-fold serial dilutions in a volume of 100 μL in an 8-well plate. Media was served as a negative control, while the diluted reference serum was added to the designated wells. Then, 100 μL of 50 × FFD_50_ CVS-11 was added into each well. The mixture was incubated for 1.5 h at 37 °C. The BSR cells were seeded at 1 × 10^5^ cells per well in 100 μL followed by incubation at 37 °C in the 5% CO_2_ incubator for 20–24 h. After incubation, the cells were fixed in 80% acetone at room temperature for 30 min and were stained with FITC-conjugated RABV N-protein at 37 °C for 1 h. After twice washes with PBS, a drop of 80% glycerol was added to each well. The number of positive microscopic fields (i.e., containing one or more infected cells) among 20 microscopic fields per well was counted under an inverted fluorescence microscope, and the percentage of positive fields (i.e., infectivity) was calculated. The 50% end-point titer of the test serum was determined using the Reed–Muench method [22]. The fluorescence values obtained from the measured serum were compared to those of a reference rabies immunoglobulin (lot 250011-201306) obtained from the National Institutes for Food and Drug Control, Beijing, China. The VNA titers from both tests were normalized and quantified in international units per millilitre.

### 2.10. Vaccine Potency Test

Vaccine potency was measured using the NIH method [23]. The potency reference standards and ABO1005 were serially 5-fold diluted (e.g., 125-fold, 625-fold, and 3125-fold) as according to the requirements of the Chinese Pharmacopoeia [23]. Kunming mice (weight 12–14 g) were vaccinated via two intraperitoneal injections administered 7 days apart. On day 7 after the booster vaccination, the mice were administered with 0.03 μL of live CVS virus via an intracranial injection at a viral dose of 30–60-fold 50% lethal dose (LD_50_). The relative potency of the vaccines was determined by calculating the ED_50_ values of the ABO1005 and reference standard (lot 250009-201909) obtained from the National Institutes for Food and Drug Control, Beijing, China.

### 2.11. Post-Exposure Prophylaxis (PEP) Study in Beagle Dogs

In total, 3 groups of Beagle dogs, each consisting of 5–6 animals, were infected intramuscularly with fifty times of a median lethal dose (LD_50_) of the RABV BD06 strain. Six hours post exposure to the BD06 strain, the Beagle dogs were immunized with either phosphate-buffered saline (PBS), an inactivated vaccine control, or an ABO1005 mRNA vaccine. In the inactivated vaccine control group, the animals received one human dose of the vaccine on days 0, 3, 7, 14, and 28. In contrast, the mRNA vaccine group was administered 15 μg per dose of ABO1005 on days 0 and 7 following virus exposure. The animals were monitored for 60 days post challenge to assess the development of rabies-specific symptoms or death, the RABV in brain tissue was confirmed using the direct fluorescent antibody test (DFA) [24,25].

### 2.12. Statistical Analysis

All data were analyzed using GraphPad Prism 9.0 software. Antibody-related data are presented as the geometric mean titer (GMT) with a 95% confidence interval (CI), while other data are presented as the mean with standard deviation (SD). The statistical significance was assessed by one-way ANOVA with Tukey’s multiple comparisons test or the Mann–Whitney *t* test. Survival data were analyzed using the log-rank test. Correlation analysis was conducted using simple linear regression. A *p*-value of ≤0.05 (*), ≤0.01 (**), and ≤0.001 (***) between the groups was considered as the degree of significant difference.

## 3. Results

### 3.1. Construction and Characterization of ABO1005

**Figure 1:**
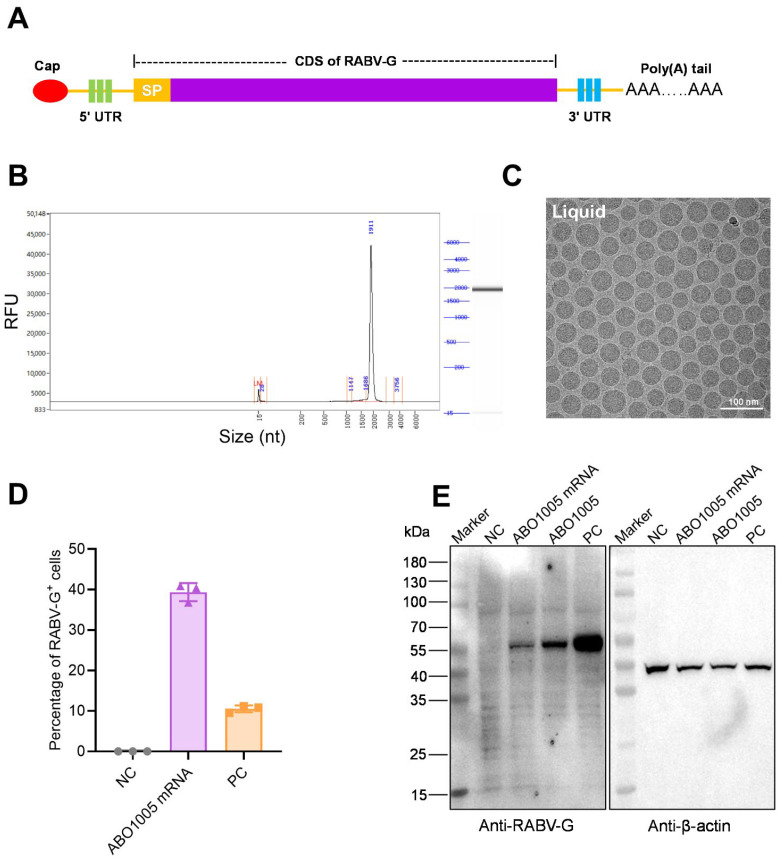
The design and characterization of ABO1005. (**A**) ABO1005 composes a 5′ Cap, a 5′ UTR, a coding sequence (CDS) of RABV-G derived from the PM strain, a 3′ UTR and a poly (**A**) tail. (**B**) The integrity of the mRNA. (**C**) The cryogenic electron microscopy (Cryo-EM) of ABO1005. (**D**) The percentage of trimer-form RABV-G-positive cells in transfected HEK293F cells. (**E**) The Western blot analysis of RABV-G expression in transfected HEK293T cells.

RABV-G serves as a single envelope protein in RABV and is responsible for the induction of VNA in vivo. In this study, we developed an LNP-encapsulated, codon-optimized mRNA formulation, designated as ABO1005. The mRNA sequence encompasses the signal peptides (SPs) and coding sequence (CDS) of the wild-type RABV-G from the Pitman-Moore (PM) strain flanked by a 5′ UTR and a 3′ UTR followed by a shorter poly (A) tail (Figure 1A). The purity of the synthetic mRNA in vitro exceeded 95% (Figure 1B). The final LNP formulations were prepared using a modified procedure based on a method previously described for mRNA vaccines [17]. The cryo-electron microscopy (cryo-EM) revealed that the average size of the mRNA-LNP was approximately 60 nm (Figure 1C).

Mature RABV-G is a transmembrane protein primarily localized on the membranes of RABV and infected cells. To investigate this characteristic, we transfected HEK293T cells with ABO1005, using pcDNA3.1-RABV-G-PM as a positive control and PBS as a negative control. In both the ABO1005 and positive control groups, the RABV-G trimer-specific antibody (RVA122-IgG1) was used to detect the expressed protein on the cells. The results showed that a strong fluorescent signal was detected on the cellular membrane (Figure 1D,E), confirming that the RABV-G protein was indeed expressed. The molecular weight of RABV-G is approximately 65 kDa [26]. Similarly, HEK293T cells transfected with ABO1005 exhibited an antibody-specific protein that concentrated around 65 kDa (Figure 1E). These findings demonstrate that ABO1005 successfully expressed RABV-G on the plasma membrane of HEK293T cells, displaying a molecular weight and trimer structure comparable to RABV-G found on the RABV membrane.

### 3.2. ABO1005 Induced Robust Humoral Responses in Mice

**Figure 2:**
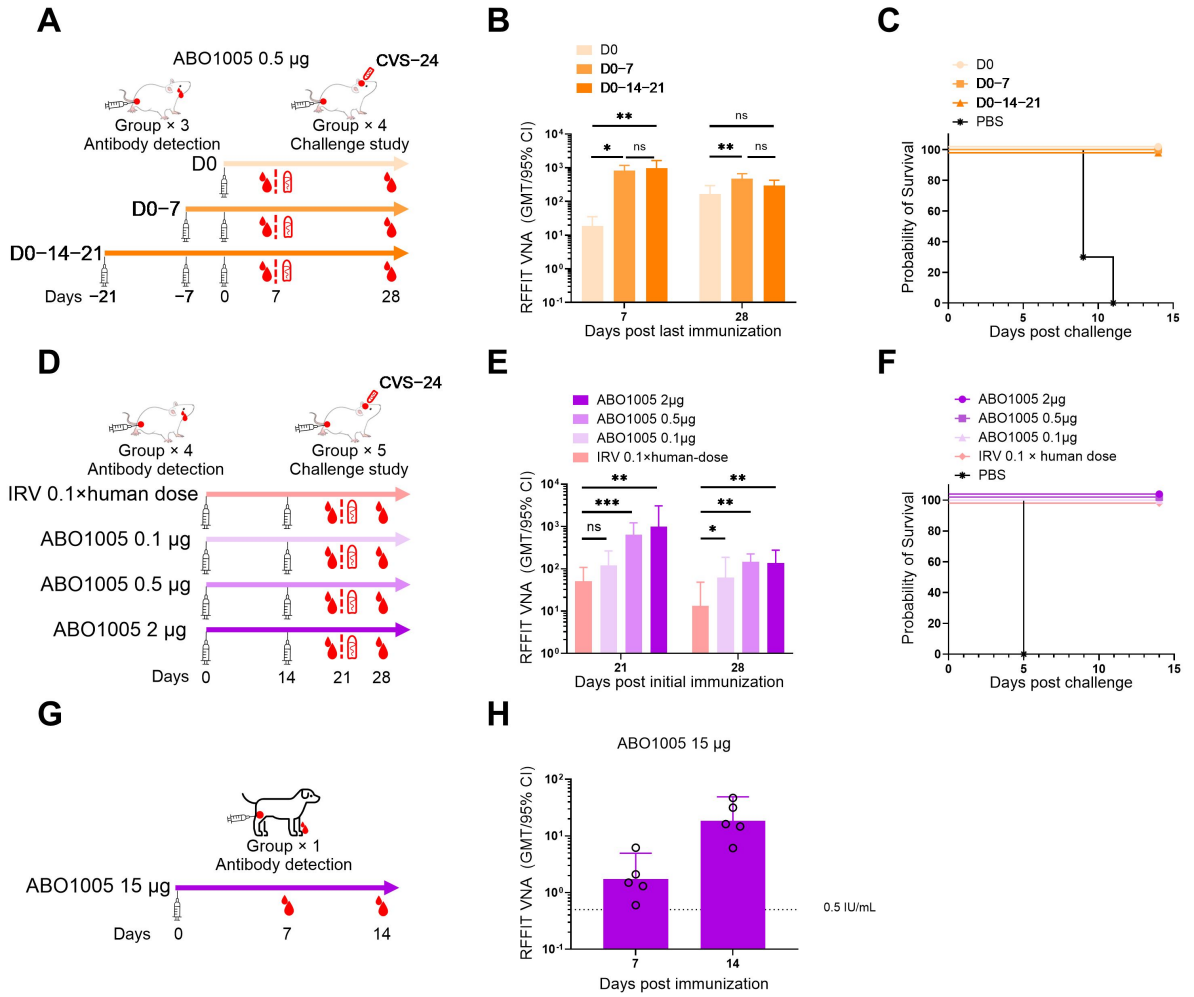
Humoral immune responses of ABO1005 in mice and dogs. (**A**) The experimental layout of the immunization regimen study. (**B**) VNA titers of the sera from mice (*n* = 10) in the regimen study. (**C**) Survival curves of the mice (*n* = 10) intracerebrally (I.C.) infected with the virulent CVS-24 strain (50 × LD_50_) in the regimen study. (**D**) The experimental layout of the dosage titration study. (**E**) VNA titers of the sera from mice (*n* = 5~9) in the dosage titration study. (**F**) Survival curves of the mice in the dosage titration study (*n* = 10). (**G**) The experimental layout of the immunogenicity study in rural dogs. (**H**) VNA titers in sera of rural dogs (*n* = 5) post I.M. immunization with a single dose of 15 μg of ABO1005. *, *p* ≤ 0.05; **, *p* ≤ 0.01; ***, *p* ≤ 0.001; ns, not significant.

Currently, individuals exposed to rabies typically require three or more visits for vaccination. Researchers are actively working to reduce the visit frequency. Neutralizing antibodies induced by rabies vaccines provide a protective effect for both animals and humans [27]. In this study, we assessed the immunogenicity of shorter vaccination regimens for 0.5 μg ABO1005 in mice (Figure 2A). On day 7 post the last immunization, the geometric mean titers (GMTs) of three-dose, two-dose and single-dose groups were 976 (367–3676), 819 (451–1906) and 19 (6–58) IU/mL, respectively. On day 28 post last immunization, the GMTs of three-dose, two-dose and single-dose groups were 293 (115–531), 470 (254–989) and 167 (47–500) IU/mL, respectively (Figure 2B). Our results demonstrated that the two-dose (D0-7) regimen was significantly more effective than the single-dose regimen on both day 7 and 28 post last immunization. However, no significant difference was observed between the two-dose and three-dose regimens.

To compare the immunogenicity of ABO1005 with that of a licensed IRV, we measured the VNA titers induced by both vaccines using a mouse model (Figure 2D). As illustrated in Figure 2E, both ABO1005 and the IRV achieved VNA titers exceeding the threshold concentration of 0.5 IU/mL recommended by the WHO. On day 21 post initial immunization, the GMTs were 997 (96–7745), 635 (158–2385), 121 (22–284) and 51 (28–119) IU/mL for 2 μg, 0.5 μg and 0.1 μg doses of ABO1005 and the IRV, respectively. On day 28 post initial immunization, the GMTs were 138 (36–488), 147 (61–360), 63 (13–380) and 13 (5–74) IU/mL for 2 μg, 0.5 μg and 0.1 μg doses of ABO1005 and the IRV, respectively. On days 21 and 28 post initial immunization, both the 2 μg and 0.5 μg doses of ABO1005 induced significantly higher VNA titers compared to the IRV. Notably, there was no significant difference in VNA titers between the 0.1 μg doses of ABO1005 and the 0.1 × human dose of the IRV on day 21, indicating that the 0.1 μg dose of ABO1005 possesses comparable immunogenicity to the 0.1 × human dose of the IRV in mice.

### 3.3. Low Dose of ABO1005 Protected Mice Against Lethal RABV Challenge

Two independent challenge studies were conducted to investigate the protective efficacy of ABO1005 in a mouse model. In one study (Figure 2C), three doses (D0-14-21), two doses (D0-7), and even a single dose of 0.5 μg ABO1005 were shown to protect mice from I.C. infection with a virulent RABV CVS-24 strain (50 × LD_50_) on day 7 after the last immunization. In contrast, all mice in the PBS group died within 11 days following virulent RABV challenge (Figure 2C). In a dose titration experiment (Figure 2F), two doses (D0-14) of 2 μg, 0.5 μg, and even 0.1 μg of ABO1005 and 0.1 × human dose of the IRV were effective in protecting mice from I.C. infection with virulent RABV. However, all mice in the PBS group died within 5 days following the virulent RABV challenge. These results demonstrate that either a single dose of 0.5 μg ABO1005 or two doses of 0.1 μg ABO1005 are sufficient to protect mice from RABV challenge.

### 3.4. ABO1005 Induced Specific T Cell Activation and Germinal Center B Cell Reaction

**Figure 3:**
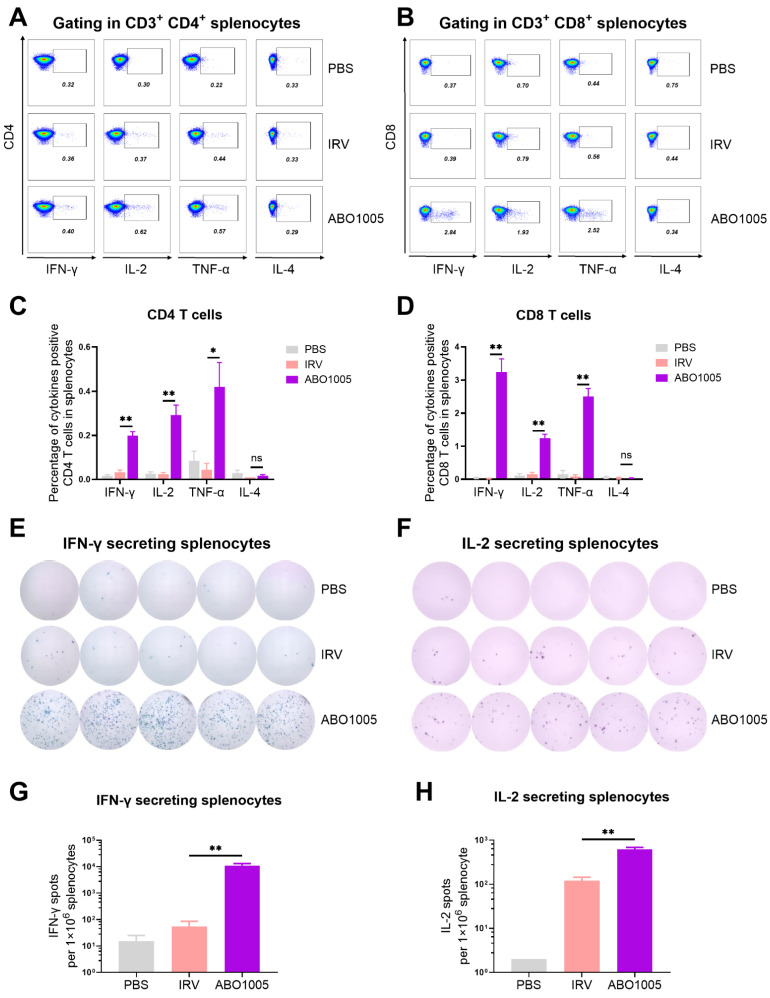
Cellular immune responses of ABO1005 in mouse model. On day 21 post initial immunization, splenic cells from mice (*n* = 5) were analyzed. (**A**) Representative pseudo-color plots of cytokine-positive CD4^+^ T cells (**A**) and CD8^+^ T cells (**B**). Statistical percentages of cytokine-positive CD4^+^ T cells (**C**) and CD8^+^ T cells (**D**). Representative IFN-γ (**E**)- and IL-2 (**F**)-secreting cell spots from five splenic samples of each group. Statistical spot-forming units (SFU) of IFN-γ (**G**)- and IL-2 (**H**)-secreting cells. *, *p* ≤ 0.05; **, *p* ≤ 0.01.

The effectiveness of rabies vaccines is conventionally assessed by measuring the secretion of neutralizing antibodies [28]. However, activated T cells, typically T helper (Th) cells and follicular helper T (Tfh) cells, play a crucial role in supporting B cell proliferation. An increased humoral response was observed in ABO1005-treated mice, which prompted our investigation into the activities of T cells following ABO1005 vaccination. As shown in Figure 3A–D, with the exception of IL-4-positive T cells, which were almost undetectable across all three groups of mice, the administration of 1.5 μg ABO1005 (0.1 × human dose, proposed) resulted in a significantly higher induction of IFN-γ-, TNF-α- and IL-2-positive CD4 and CD8 T cells compared to the IRV (0.1 × human dose), as detected by FACS (Figure 3A–D). Furthermore, ELISpot assays confirmed that the number of IFN-γ- and TNF-α-secreting cells in the ABO1005 group was markedly greater than that in the IRV group (Figure 3E–H). Hence, in the mouse model, ABO1005 induced a significantly higher level of specific Th1 cells compared to the IRV.

**Figure 4:**
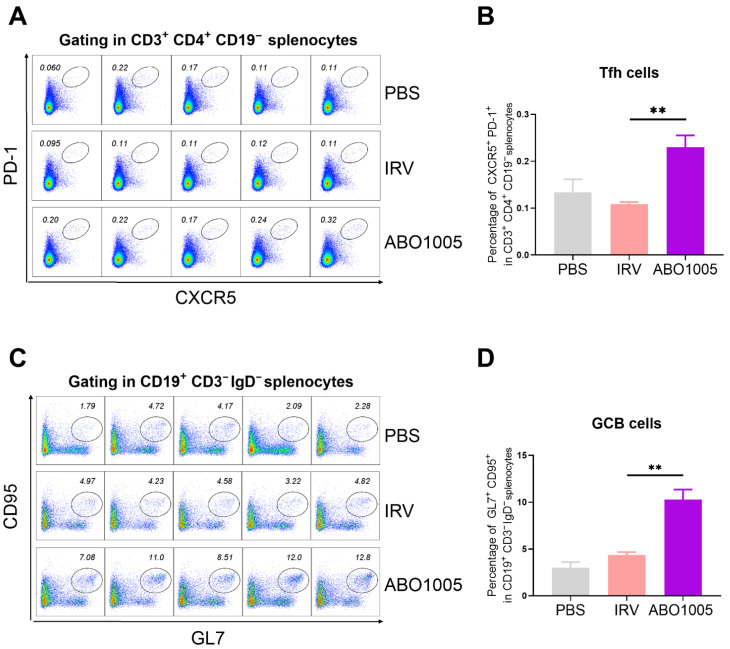
ABO1005 induces proliferation of Tfh and GCB in mouse spleens. On day 21 post initial immunization, splenic cells from mice (*n* = 5) were analyzed. Representative pseudo-color plots (**A**,**C**) and statistical percentages (**B**,**D**) of Tfh and GCB cells. **, *p* ≤ 0.01.

The quality of germinal centers (GCs) is directly regulated by Tfh cells, which provide essential signals for the differentiation and proliferation of GC B cells (GCB). As shown in Figure 4A–D, in the spleens of immunized mice, ABO1005 induced significantly higher percentages of both Tfh and GCB cells compared to the IRV in the mouse model. These results indicate that ABO1005 can induce the potent activation of T cells and promote the proliferation of Tfh and GCB cells.

### 3.5. ABO1005 Elicited Long-Lasting Neutralizing Antibodies and Immunogenicity Was Not Affected by Pre-Administration of Hyperimmune Serum

**Figure 5:**
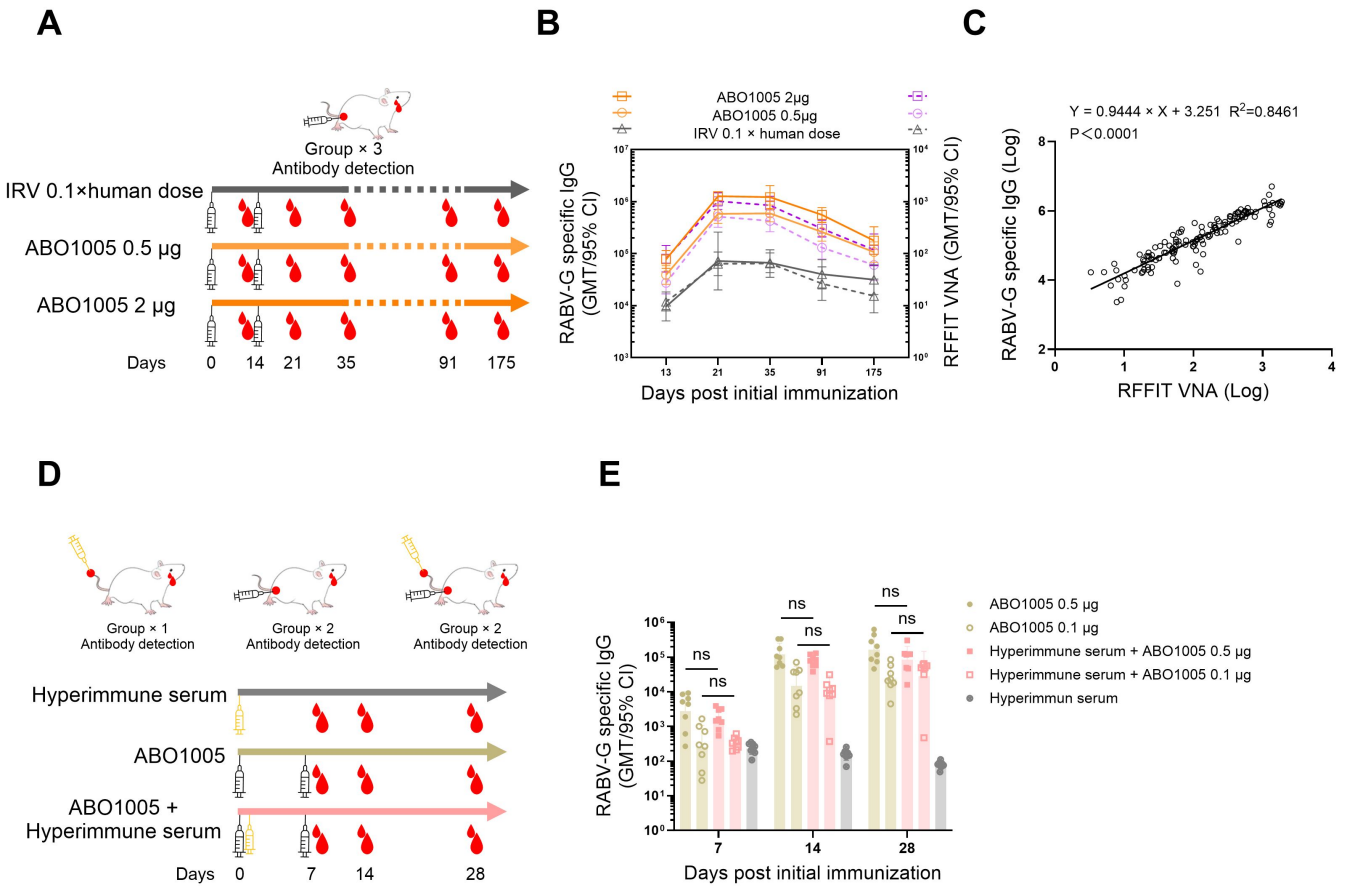
Duration of humoral immunity and effect of pre-administration with rabies-specific immunoglobulin on immunogenicity of ABO1005. (**A**) Experimental layout of duration study. (**B**) Duration of humoral immunity in mice. (**C**) Correlation analysis between RABV-specific IgG titers and VNA titers (*n* = 137). (**D**) Experimental layout of study on effect of pre-administration with rabies specific immunoglobulin. Groups of mice (*n* = 8) were injected I.V. with 2 IU (VNA titers) of rabies hyperimmune serum diluted in PBS buffer or PBS alone, followed by I.M. immunization with two doses of ABO1005. One group (*n* = 8) of mice received only I.V. injection of rabies hyperimmune serum. (**E**) RABV-specific IgG titers in sera were measured using ELISA. ns, not significant.

The duration of specific antibody induction by vaccines in mice was compared between ABO1005 and the IRV. We monitored both VNA- and RABV-G-specific IgG titers over a period of 175 days (Figure 5A). Figure 5B illustrates that both the 2 μg and 0.5 μg doses of ABO1005 induced higher VNA and IgG titers compared to the IRV, with the temporal trend of IgG titers mirroring that of VNA titers. Further analysis revealed a strong correlation between the titers of VNA and IgG, as shown in Figure 5C (*κ* = 0.9444, *n* = 137; *r* = 0.8461, *p* < 0.0001).

The WHO recommends the administration of both rabies immunoglobulin and vaccines for category III exposure [29]. However, rabies VNAs may bind to the antigens in rabies vaccines, potentially weakening the immune response following vaccine administration. To investigate this interference between ABO1005 and mouse-derived rabies hyperimmune serum, mice were injected I.M. with two doses of ABO1005 on days 0 and 7, with or without a single tail-vein injection of hyperimmune serum (2 IU) on day 0. The RABV-G-specific IgG titers were measured on days 7, 14, and 28 following the initial injection of ABO1005 (Figure 5D). The results indicated a significant increase in IgG titers following the first immunization from day 7 to day 28, and no significant differences were observed in IgG titers between the groups receiving ABO1005 alone and those receiving ABO1005 in conjunction with hyperimmune serum (Figure 5E).

### 3.6. Lyophilized ABO1005 Protected Beagle Dogs in a Post-Exposure Prophylaxis Model

**Figure 6:**
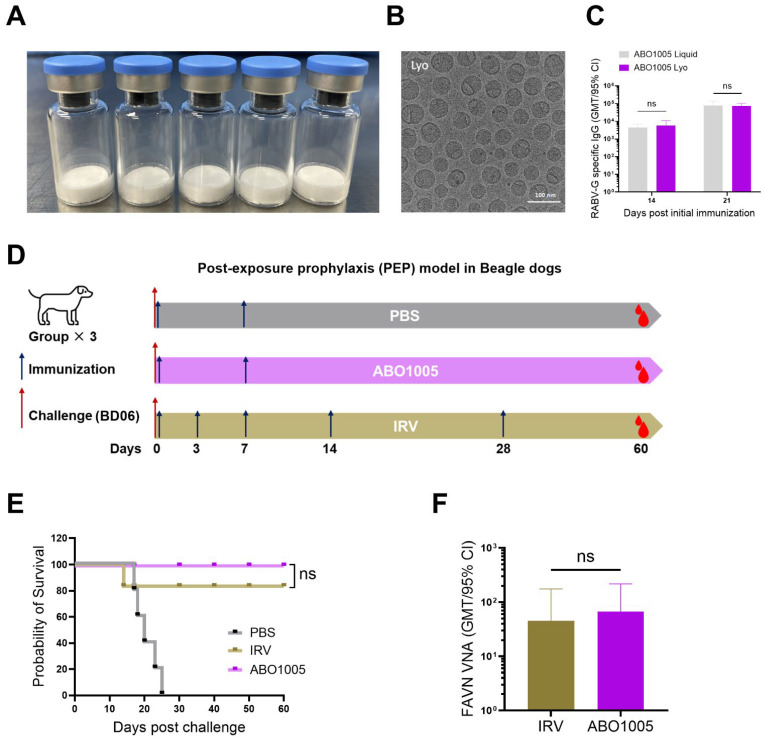
Lyophilized ABO1005 protected Beagle dogs in PEP model. (**A**) The images of lyophilized vials containing ABO1005. (**B**) Spherical nanoparticles of lyophilized ABO1005. (**C**) The comparison of immunogenicity between liquid and lyophilized ABO1005 (0.12 μg per dose on days 0 and 14) in mice (*n* = 8). (**D**) The experimental layout of the PEP study. (**E**) Survival curves of dogs (*n* = 6). (**F**) VNA titers of the survived dogs (*n* = 5~6) on day 60 post initial immunization (challenge). ns, not significant.

The physicochemical properties of mRNA-LNPs can be effectively preserved through lyophilization technology [30]. In fact, most commercially available rabies vaccines are freeze-dried and typically have a shelf life of 3 to 4 years, which poses challenges for the market entry of liquid or frozen formulations. Consequently, we developed the lyophilized ABO1005 formulation and compared its immunogenicity with that of the liquid formulations. As anticipated, the lyophilized ABO1005 exhibited a uniform and dense white cake appearance (Figure 6A). The morphology of lyophilized particles was captured using Cryo-EM (Figure 6B). The size observed in the Cyro-EM picture is approximately 65 nm, and bleb structures were noted on the lyophilized LNP, likely resulting from the physical stress induced by vapor sublimation. On days 14 and 21, no significant differences were observed in the RABV-G-specific IgG titers or VNA titers in mice between the lyophilized ABO1005 and the liquid ABO1005 (Figure 6C and Figure S1). The results indicate that the physical properties and biological activity of the ABO1005 formulation can be effectively preserved through lyophilization.

Rabies vaccines for human are primarily administered as post-exposure prophylaxis (PEP). The speed of antibody production is critical for vaccine efficacy. We initially assessed the rabies VNA titers in rural dogs on days 7 and 14 following immunization. The results demonstrated that VNA titers achieved 100% seroconversion by day 7 and continued to rise by day 14 (Figure 2H). To further investigate the protective efficacy of lyophilized ABO1005, we conducted a PEP study using Beagle dogs as previously described [3,31]. Initially, three group of Beagle dogs were infected I.M. with the street strain BD06 (50 × LD_50_). Six hours after RABV injection, one group of Beagle dogs were vaccinated I.M. with two doses (D0-7) of 15 μg ABO1005, and another group of animals received five doses (D0-3-7-14-28) of 1 × human dose IRV, respectively. The third group received PBS as a control (Figure 6D). As expected, all Beagle dogs in the control group died within 25 days. The final survival rate of the ABO1005 group was 100% (6/6). In contrast, 83.3% (5/6) of the Beagle dogs in the IRV group survived by the end of the study (Figure 6E). On day 60, VNA titers of all surviving Beagle dogs were analyzed using FAVN, and no significant difference was observed between the ABO1005 group (67.0 IU/mL) and the IRV group (45.2 IU/mL) (Figure 6F). Collectively, two doses (D0-7) of 15 μg lyophilized ABO1005 provided complete protection against the BD06 strain challenge in dogs and demonstrated efficacy comparable to that of the five doses (D0-3-7-14-28) of the commercial IRV.

### 3.7. ABO1005 Has a Potency That Meets the Standards for Human Vaccine Release

The potency of ABO1005 was measured using the potency determination method for human rabies vaccines (NIH method), as specified in the 2020 edition of the Chinese Pharmacopoeia. The potency of ABO1005 in human doses of 15 μg was 8.85 IU/dose (Table 1). According to the Chinese Pharmacopoeia, the required potency for human rabies vaccines during shelf-life and at release is ≥2.5 IU/dose and ≥4.0 IU/dose, respectively. The potency of ABO1005 at 15 μg/dose was 8.85 IU/dose, which meets the standard for lot release.

### 3.8. Lyophilized ABO1005 Vaccine Maintained Long-Term Stability

To date, the two approved COVID-19 mRNA vaccines Spikevax and Comirnaty both required frozen storage to prevent nucleic acid hydrolysis and degradation. Lyophilization is believed to improve stability by removing water from the formulation.

**Figure 7:**
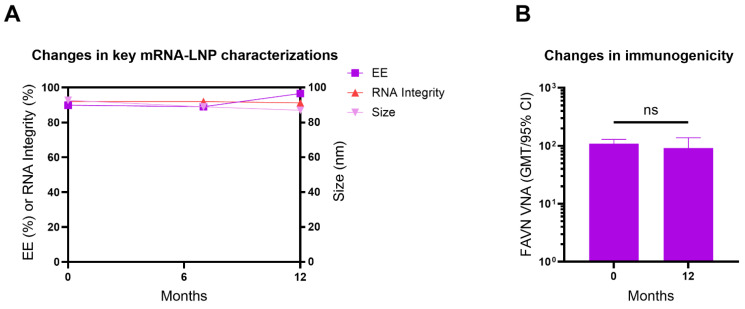
The lyophilized ABO1005 vaccine exhibited long-term stability. (**A**) The size distribution, encapsulation efficiency and mRNA integrity of the cargo encapsulated in lyophilized ABO1005. (**B**) At storage after months 0 and 12, the immunogenicity changes in lyophilized ABO1005 were determined in mice. ns, not significant.

This study investigated the particle size, encapsulation efficiency (EE), and integrity of mRNA in the lyophilized ABO1005 at months 0, 7 and 12. The results indicated that the vaccine remained stable for at least 12 months when stored at 2–8 °C (Figure 7A), and further investigations into the long-term stability of the lyophilized vaccine are ongoing. The immunogenicity of lyophilized ABO1005 was evaluated in mice at month 0 and month 12, respectively. The results demonstrated that the VNA titers were comparable at the tested time points, indicating that the immunogenicity remained unchanged after the lyophilized vaccine was stored for at least twelve months (Figure 7B).

## 4. Discussion

Inactivated rabies vaccines are generally effective in preventing rabies; however, individuals who experience severe exposure (category III) often rely on passive immune protection through the administration of hyperimmune sera or antibodies. Inactivated vaccines alone have proven inadequate in providing sufficient protection against lethal challenges in dogs [32]. Each year, fatalities occur despite the combined administration of rabies vaccines and passive immune products, highlighting the urgent need to enhance the protective efficacy of rabies vaccines [33]. This study demonstrates that, in the absence of passive immunity, two doses of the mRNA rabies vaccine can achieve 100% protection in a post-exposure prophylaxis model in dogs, whereas conventional inactivated vaccines confer only 83% protection (Figure 6E). These findings suggest that the mRNA rabies vaccine offers superior protection compared to commercially available inactivated vaccines.

Previous studies have identified RABV-G as the primary antigen target for rabies vaccines [34], and optimizing the coding sequence for mammalian cells significantly enhances antigen expression, thereby improving immunogenicity [35]. Notably, the trimeric pre-fusion form of the G-protein is considered the optimal immunogen due to its surface displaying the most prominent VNA epitopes [2]. To date, no successful methods have been identified for producing a stable, soluble pre-fusion trimeric recombinant RABV-G protein [13]. We constructed a codon-optimized mRNA that expresses RABV-G on the external membrane of human-derived cells, which was recognized by the trimer-specific antibody RVA122-IgG1 (Figure 1D).

The inactivated rabies vaccine (IRV) requires multiple doses to achieve sufficient levels of rabies VNA. These regimens are not only time-consuming but also expensive, posing significant challenges in compliance for completing the whole vaccination in developing countries that experience high rates of rabies-related deaths. In 2018, the WHO’s third expert consultation on rabies recommended both the four-dose Zagreb regimen (21 days) and the four-dose ACIP regimen (14 days) and excluded the five-dose Essen regimen (28 days) [29]. Currently, several clinical trials are underway to investigate novel vaccines, adjuvants, and vaccination regimens [10]. Cadila Pharma has launched a three-dose (1-1-1, D0-3-7) recombinant rabies vaccine, Thrabis^®^, in India [34]. Yisheng Biopharma is developing a five-dose (2-2-1, D0-3-7) rabies vaccine that incorporates the PIKA adjuvant, demonstrating non-inferior immunogenicity compared to a commercial vaccine using the ACIP regimen in healthy adults [36]. CureVac AG conducted proof-of-concept clinical trials of mRNA-based vaccines encoding RABV-G (CV7201 and CV7202) in healthy adults [11]. However, CureVac AG stopped further developing these two mRNA vaccines due to a concern about the potency and safety of their mRNA vaccine candidates. In the current study, the two doses of ABO1005 administered in 7 days exhibited superior immunogenicity compared to a single dose, and this regimen demonstrated equivalent immunogenicity to the three-dose regimen of ABO1005 administered within 21 days. Under the same two-dose regimen within 14 days, a low dose (0.5 μg) of ABO1005 achieved a significantly higher VNA titer than 0.1 × human dose IRV (Figure 2E). These findings indicated that the rabies mRNA vaccine ABO1005 has superior immunogenicity to the IRV and support the further development of an accelerated regimen utilizing ABO1005.

Recently, several preclinical studies on rabies mRNA vaccines have been reported [37]. Among these, Li et al. reported that, in a mouse challenge study, a lyophilized mRNA-LNP rabies vaccine, LVRNA001, provided 58% and 78% protection following single-dose immunization at 1 μg and 5 μg, respectively [8]. In contrast, in the current study, ABO1005 achieved 100% protection at a single dose of 0.5 μg (Figure 2C). Two doses (D0-14) of 0.625 μg of LVRNA001 resulted in 80% protection against the challenge with the CVS strain on day 21, while 0.1 μg of ABO1005 achieved 100% protection under the same immunization regimen (Figure 2F). Despite the fact that the studies were carried out under different experiment settings, the data indicated differences in the immunogenicity of the two vaccines. However, both LVRNA001 and ABO1005 achieved 100% protection in post-exposure immunization experiments conducted in dogs. This finding suggests that, in addition to humoral immunity, mRNA-mediated cellular immunity could contribute to vaccine protection [38].

In clinical applications, rabies vaccines are frequently administered in conjunction with hyperimmune sera, which can provide a certain level of pre-existing immunity to the host. Research suggested that pre-existing antibodies may accelerate the clearance of antigens expressed by mRNA vaccines, potentially diminishing their immunogenicity [39]. In the present study, mice were administered hyperimmune sera via the tail vein prior to intramuscular immunization with ABO1005. The results indicated that the pre-existing antibodies conferred by hyperimmune sera did not affect the rate of antibody onset following the initial immunization or the booster effect of the subsequent immunization with ABO1005 (Figure 5E). Although this study did not evaluate neutralizing antibodies, it can be inferred from the correlation between binding antibodies and neutralizing antibodies (Figure 5C) that pre-existing specific antibodies do not negatively impact the neutralizing antibody response elicited by ABO1005.

The durability of the immunogenicity of mRNA vaccines is a common concern, particularly in light of the observed decline in the protective efficacy of Moderna’s marketed RSV vaccine, mResvia, which decreased from approximately 80% at four months post immunization to around 56% at twelve months post immunization [40]. In our study, at a human dose of 15 μg/dose, the potency of ABO1005 was 8.85 IU/dose, which is substantially higher than the standard for the lot release of rabies vaccines for current human use. This indicates that ABO1005 provides an acceptable protective effect. And mice that received two doses of the mRNA vaccine maintained serum neutralizing antibodies above a titer of 30 IU/mL for approximately six months, demonstrating significantly better persistence compared to the marketed inactivated vaccine (Figure 5B). Notably, mResvia is administered as a single-dose immunization, whereas ABO1005 was designed for a two-dose regimen, with the booster dose facilitating long-lasting immunogenicity.

Currently, most mRNA-LNP-based vaccines require frozen storage, which limits their global distribution. Lyophilized mRNA-LNP formulations have the potential to overcome these challenges and extend the shelf-life of vaccines. However, the freezing and dehydration processes can destabilize the particles, leading to concomitant cargo leakage; thus, the development of a robust LNP lyophilization process remains challenging [41,42]. The study data demonstrated that lyophilized ABO1005 not only maintains proper physicochemical properties but also exerts strong immunogenicity following 2–8 °C storage for at least twelve months. Further, the lyophilized ABO1005 achieved a 100% survival rate in Beagle dogs in the PEP model. We believe this work represents a significant advancement in the field and ABO1005 could potentially serve as an effective rabies vaccine candidate.

Certainly, the rabies mRNA vaccine may encounter challenges beyond stability when applied in real-world scenarios. With approximately 5 billion doses of COVID-19 mRNA vaccines administered, concerns such as myocarditis and the risk of genomic integration have emerged. The link between the administration of COVID-19 mRNA vaccines and myocarditis in young adults was supported by some clinical data, although the incidence remains extremely low and may be spike protein-specific [43,44]. The risk of genomic integration has been indicated through in vitro experiments by over-expressing the long interspersed nucleotide element-1 (LINE-1) reverse transcriptase [45], but clinical evidence is lacking, and some researchers hold a contradictory viewpoint [46]. Therefore, we believe that the risk of genomic integration from mRNA vaccines is low. Nonetheless, the potential risks still require validation in human trials.

## 5. Conclusions

In summary, a lyophilized rabies mRNA vaccine candidate has been shown to produce both robust humoral and cellular immune responses and protective efficacy in preclinical studies. The lyophilized mRNA vaccine was stable at 2–8 °C storage for at least twelve months. These findings support the further development of lyophilized ABO1005 as an effective rabies vaccine, thereby facilitating the application of lyophilization technology in the development of mRNA vaccines targeting other infectious pathogens or cancers, particularly in the context of global health initiatives and vaccine accessibility in remote areas.

## Figures and Tables

**Table 1:** Potency of ABO1005 as measured using NIH method.

Group	Dilution	Survival		Accumulated			Result	
		Death	Live	Death	Live	Death Rate	Log ED_50_	Potency
PBS	\	16	0	\	\	100%	\	\
9th Std ^1^	×125	3	13	3	23	11.5%	2.75	11.4 IU/mL
	×625	8	8	11	10	52.3%		
	×3125	14	2	25	2	92.6%		
ABO1005	×125	0	16	0	26	0%	2.94	8.85 IU/dose (0.5 mL)
	×625	6	10	6	10	37.5%		
	×3125	16	0	22	0	100%		

^1^: 9th Chinese National Standard for Human Rabies Vaccines (labeled potency: 11.4 IU/mL).

## Data Availability

The datasets presented in this study can be found in online repositories. The names of the repository/repositories and accession number(s) can be found below: DQ099525.1 (GenBank).

## Supplementary Materials

The following supporting information can be downloaded at https://www.mdpi.com/article/10.3390/vaccines13070743/s1, Figure S1: Virus-neutralizing antibody of lyophilized ABO1005 and its liquid counterpart; Figure S2: Original images for Cryo-EM.; Figure S3: Original images for WB.
