# Supplementary material for: Immunogenicity, Efficacy and Twelve-Month Storage Stability Studies of a Lyophilized Rabies mRNA Vaccine
Source: Vaccines (Basel). 2025 Jul 10;13(7):743. doi: 10.3390/vaccines13070743 (PMC12299307; doi:10.3390/vaccines13070743)
Supplement: Supplementary file 1 [file vaccines-13-00743-s001.zip › Figure S1.pdf]

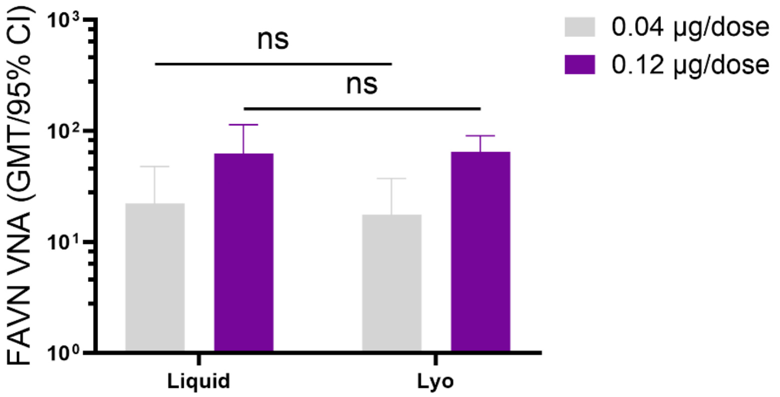

Figure S1. Virus neutralizing antibody of lyophilized ABO1005 and its liquid counterpart. Groups of KM mice (n = 8) were I.M. immunized with 0.04 µg or 0.12 µg of ABO1005 per dose on day 0 and day 14. Virus neutralizing antibody (VNA) titers were measured on day 21. ns, not significant.
