# Supplementary figures and images for: Immunogenicity, Efficacy and Twelve-Month Storage Stability Studies of a Lyophilized Rabies mRNA Vaccine
Source: Vaccines (Basel). 2025 Jul 10;13(7):743. doi: 10.3390/vaccines13070743 (PMC12299307; doi:10.3390/vaccines13070743)

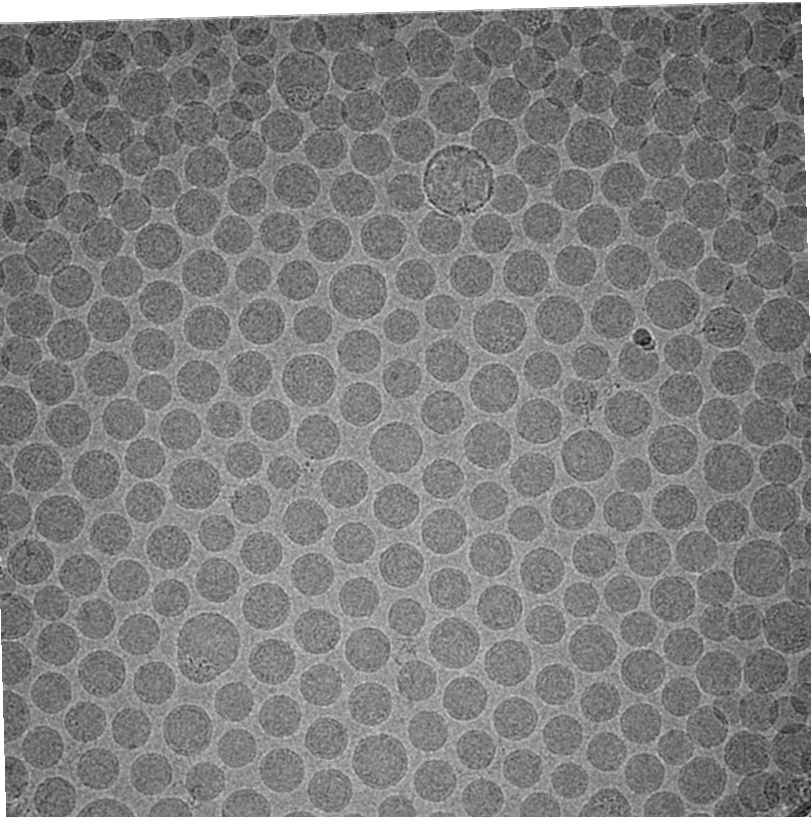

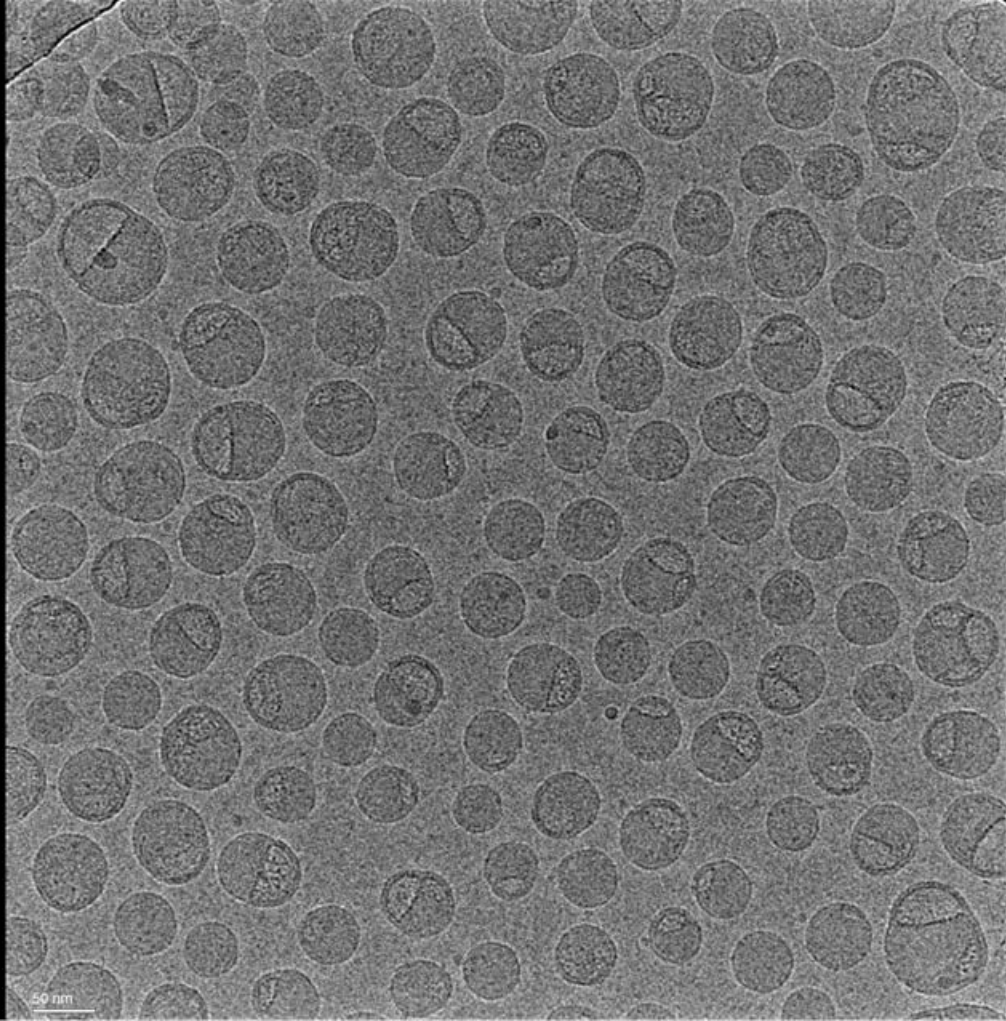

Supplement: Supplementary file 1 [file vaccines-13-00743-s001.zip › Figure S2 Original images for Cryo-EM.pdf]

anti-rabies G

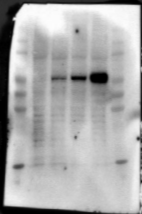

anti-actin

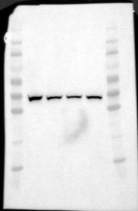

Supplement: Supplementary file 1 [file vaccines-13-00743-s001.zip › Figure S3 Original images for WB.pdf]
